# Clinical outcomes of intravenous immunoglobulin therapy in COVID-19 related acute respiratory distress syndrome: a retrospective cohort study

**DOI:** 10.1186/s12890-021-01717-x

**Published:** 2021-11-08

**Authors:** Husain S. Ali, Moustafa S. Elshafei, Mohamed O. Saad, Hassan A. Mitwally, Mohammad Al Wraidat, Asra Aroos, Nissar Shaikh, Dore C. Ananthegowda, Mohamed A. Abdelaty, Saibu George, Abdulqadir J. Nashwan, Ahmed S. Mohamed, Mohamad Y. Khatib

**Affiliations:** 1 Department of Medical ICU/Medicine, Hamad General Hospital, P.O. Box 3050, Doha, Qatar; 2 Intensive Care Unit, Hazm Mebaireek General Hospital, Doha, Qatar; 3 Department of Pharmacy, Al Wakra Hospital, Al Wakrah, Qatar; 4 Department of Surgical ICU, Hamad General Hospital, Doha, Qatar

**Keywords:** Acute respiratory distress syndrome, COVID-19, ICU mortality, Intravenous immunoglobulin, Mechanical ventilation

## Abstract

**Background:**

Intravenous immunoglobulin (IVIG) has been used as an immunomodulatory therapy to counteract severe systemic inflammation in coronavirus disease 2019 (COVID-19). But its use in COVID-19 related acute respiratory distress syndrome (ARDS) is not well established.

**Methods:**

We conducted a retrospective analysis of electronic health records of COVID-19 patients admitted to intensive care units (ICUs) at Hazm Mebaireek General Hospital, Qatar, between March 7, 2020 and September 9, 2020. Patients receiving invasive mechanical ventilation for moderate-to-severe ARDS were divided into two groups based on whether they received IVIG therapy or not. The primary outcome was all-cause ICU mortality. Secondary outcomes studied were ventilator-free days and ICU-free days at day-28, and incidence of acute kidney injury (AKI). Propensity score matching was used to adjust for confounders, and the primary outcome was compared using competing-risks survival analysis.

**Results:**

Among 590 patients included in the study, 400 received routine care, and 190 received IVIG therapy in addition to routine care. One hundred eighteen pairs were created after propensity score matching with no statistically significant differences between the groups. Overall ICU mortality in the study population was 27.1%, and in the matched cohort, it was 25.8%. Mortality was higher among IVIG-treated patients (36.4% vs. 15.3%; sHR 3.5; 95% CI 1.98–6.19; *P* < 0.001). Ventilator-free days and ICU-free days at day-28 were lower (*P* < 0.001 for both), and incidence of AKI was significantly higher (85.6% vs. 67.8%; *P* = 0.001) in the IVIG group.

**Conclusion:**

IVIG therapy in mechanically ventilated patients with COVID-19 related moderate-to-severe ARDS was associated with higher ICU mortality. A randomized clinical trial is needed to confirm this observation further.

**Supplementary Information:**

The online version contains supplementary material available at 10.1186/s12890-021-01717-x.

## Background

Coronavirus disease 2019 (COVID-19) is a highly infectious acute respiratory disease caused by a novel coronavirus, subsequently named severe acute respiratory syndrome coronavirus 2 (SARS-CoV-2) [1]. The first human cases of COVID-19 were reported in Wuhan City, China, in December 2019. Since then, the disease has rapidly spread worldwide, with World Health Organization (WHO) formally declaring it a pandemic on March 11, 2020 [2].

Over 4.8 million deaths have been reported worldwide due to COVID-19 [3]. The leading cause of death is respiratory failure due to acute respiratory distress syndrome (ARDS). In addition, almost half of the patients with COVID-19 receiving invasive mechanical ventilation died, based on the case fatality rates reported in a recent meta-analysis [4]. There is increasing evidence that a hyperinflammatory response to SARS-CoV-2 contributes to disease severity and death in COVID-19. Patients with severe disease have increased serum levels of proinflammatory cytokines such as interleukin (IL)-1, IL-2, IL-6, tumor necrosis factor (TNF)-α, and interferon (IFN)-γ [5, 6]. Therefore, an effective therapy that modulates inflammatory response, prevents clinical deterioration and improves mortality is urgently needed.

Intravenous immunoglobulin (IVIG) is a blood product prepared from the serum pooled from thousands of healthy donors. The main component of IVIG is the serum IgG fraction with traces of IgA and IgM. IVIG exerts an immunomodulatory action, involving both innate (phagocytic leukocytes, natural killer cells, and cytokines) and adaptive (B cells, T cells, and antibodies) immunity [7]. It has been successfully used to treat dermatomyositis, Guillain–Barre syndrome, immune cytopenia, post-bone marrow transplantation, vasculitis, and Kawasaki disease [8]. Due to its anti-inflammatory effect, IVIG may suppress the hyperactive immune response associated with severe COVID-19 pneumonia and improve clinical outcomes. However, only a few studies with inconsistent results have investigated the use of IVIG in critically ill SARS-CoV-2 infected patients [9, 10]. Moreover, none of the available literature reports outcomes of IVIG therapy in COVID-19 related ARDS. This prompted us to conduct a retrospective study to evaluate the efficacy and safety of IVIG in COVID-19 pneumonia patients requiring invasive mechanical ventilation for moderate-to-severe ARDS.

## Methods

### Study population, design, and setting

We retrospectively analyzed electronic health record data of patients admitted to ICUs at Hazm Mebaireek General Hospital, Qatar, between March 7, 2020 and September 9, 2020. The Medical Research Center (MRC) at Hamad Medical Corporation, Qatar, approved this study and waived the requirement for informed consent (protocol ID MRC-01-20-853). Inclusion criteria were: COVID-19 positivity as determined by reverse-transcription polymerase chain reaction (RT-PCR) of nasopharyngeal swabs, age above 18 years, respiratory failure requiring invasive mechanical ventilation, and moderate-to-severe ARDS (PaO_2_/FiO_2_ ≤ 200 mm Hg) as defined by the Berlin criteria [11]. Patients who received IVIG for indications other than COVID-19 related ARDS or had cardiac arrest before ICU admission were excluded. The study population was divided into two groups based on receiving routine care or IVIG plus routine care during their ICU stay. All patients received steroid and antiviral therapy as per the hospital's policy for COVID-19 management unless contraindicated. Prophylactic-, intermediate- or therapeutic-dose of anticoagulation was administered based on the patient's risk stratification as low-, intermediate- or high-risk for thromboembolic disease (anticoagulation protocol in Additional file 1: Fig. S1). IVIG was given to patients at the treating physician's discretion if there was a persistent increase in oxygen requirement and worsening laboratory parameters (C-reactive protein and serum ferritin level), suggestive of disease progression. The IVIG treatment group received a minimum one dose of 0.4 g/kg of IVIG. Further doses of IVIG were given on consecutive days, to a maximum of 5 doses, based on the treating physician's decision. Patients were closely monitored for immediate adverse effects like skin rash, arrhythmias, hypotension, and anaphylaxis.

### Data collection

Baseline data were collected at the time of ICU admission. Information collected were demographic characteristics, comorbid conditions, blood test results, PaO_2_/FiO_2_ ratio, vasopressor use, sequential organ failure assessment (SOFA) score, immune-modulating, and antiviral drugs received.

### Outcomes

The primary outcome studied was all-cause ICU mortality. Secondary outcomes included ventilator-free days and ICU-free days at day-28, and incidence of acute kidney injury (AKI) defined by the Kidney Disease Improving Global Outcomes (KDIGO) criteria as an increase in serum creatinine level by ≥ 0.3 mg/dl (≥ 26.5 μmol/L) within 48 h or increase in serum creatinine to ≥ 1.5 times baseline [12]. Sub-group analysis was done for patients based on PaO_2_/FiO_2_ ratio, serum ferritin level, time from ICU admission to IVIG therapy, and doses of IVIG received.

### Statistical analysis

We used propensity score matching to account for the non-random treatment allocation and to adjust for confounders. Propensity scores were generated based on age, sex, body mass index (BMI), comorbidities, SOFA score, PaO_2_/FiO_2_ ratio, baseline laboratory results, and other drug therapies. The propensity score overlap was assessed graphically. One-to-one matching with a caliper width of 0.2 of the standard deviation of the logit of the propensity score and no replacement was used to select a matching control group [13]. The post-matching covariate balance was assessed using the standardized differences. The primary outcome was compared using survival analysis. We included the treatment with IVIG as a discrete time-dependent covariate to minimize the risk of immortal time bias. Because the discharge from ICU does not fulfill the assumption of non-informative censoring, Fine-and-Gray's competing risk model was used for the primary outcome with ICU discharge as a competing event. The treatment effect on the primary outcome was described as a sub-distribution hazard ratio (sHR) with a 95% confidence interval. A robust variance estimator was used to account for correlations resulting from matching. Multiple imputation procedure was used to deal with missing data (BMI, seven values; D-dimer, nine values; and ferritin, three values). *P* values of less than 0.05 were considered statistically significant. All statistical analyses were conducted using Stata/MP 16.0 for Windows.

## Results

### Characteristics of patients

**Fig. 1:**
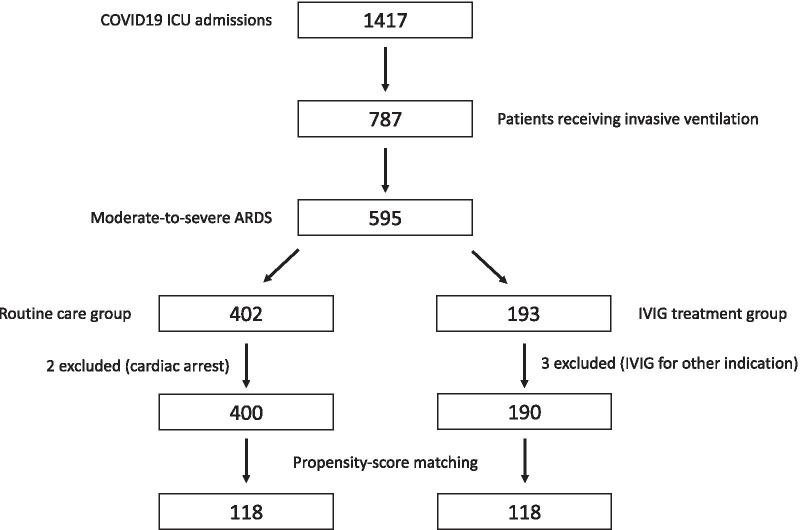
Flow chart showing selection of patients. COVID19, corona virus disease 2019; ICU intensive care unit; ARDS, acute respiratory distress syndrome; IVIG, intraveneous immunoglobulin

During the study period (between March 7, 2020 and September 9, 2020), 1417 patients were admitted to ICUs at Hazm Mebaireek General Hospital with the diagnosis of COVID-19. Seven hundred eighty-seven patients received invasive mechanical ventilation, of which 595 (75.6%) had moderate-to-severe ARDS. We excluded three patients who received IVIG for other indications from the treated group and two patients admitted to the ICU post-cardiac arrest from the control group. The remaining cohort of 590 patients comprised 190 treated with IVIG and 400 in the routine care group (Fig. 1).

A comparison between unmatched and propensity score-matched groups is shown in Table 1. Before matching, the patients in the IVIG group were older, had a higher prevalence of hypertension, dyslipidemia, and hemodialysis, had lower PaO_2_/FiO_2_ ratio, lesser vasopressor use, lower SOFA score, raised alanine transaminase (ALT), more use of dexamethasone and lesser use of methylprednisolone, hydrocortisone, tocilizumab, lopinavir-ritonavir, and oseltamivir. In the IVIG group, the median time from ICU admission to initiation of IVIG therapy was 6.28 days (IQR 2.1–11.9 days). The median cumulative dose of IVIG received was 150 g (IQR 105–235 g), and the median number of doses received was 4 (IQR 3–5).

**Table 1:** Patient characteristics

Variable	Total (n = 590)	Unmatched			Matched		
		Routine care (n = 400)	IVIG (n = 190)	*P* value	Routine care (n = 118)	IVIG (n = 118)	*P* value
Age (years)	53 (42–62)	51 (41.5–61)	56.5 (47–65)	< 0.001	52 (42–60)	53.5 (44–60)	0.36
*Gender*							
Male	555 (94.1%)	377 (94.2%)	178 (93.7%)	0.79	112 (94.9%)	112 (94.9%)	1.00
Female	35 (5.9%)	23 (5.8%)	12 (6.3)		6 (5.1%)	6 (5.1%)	
BMI (kg/m^2^)	27.2 (24.2–30.4)	26.9 (24.2–30.7)	27.6 (24.7–30.1)	0.50	26.7 (23.9–30.1)	27.69 (24.7–29.4)	0.54
*Co-morbidities*							
Diabetes mellitus	288 (48.8%)	198 (49.5%)	90 (47.4%)	0.63	51 (43.2%)	55 (46.6%)	0.60
Hypertension	290 (49.2%)	184 (46.0%)	106 (55.8%)	0.03	59 (50.0%)	58 (49.2%)	0.9
Dyslipidemia	68 (11.5%)	38 (9.5%)	30 (15.8%)	0.03	10 (8.5%)	12 (10.2%)	0.65
Coronary artery disease	77 (13.1%)	45 (11.3%)	32 (16.8%)	0.06	12 (10.2%)	14 (11.9%)	0.68
Chronic kidney disease	58 (9.8%)	44 (11.0%)	14 (7.4%)	0.17	4 (3.4%)	5 (4.2%)	1.00
Hemodialysis	33 (5.6%)	0 (0%)	33 (17.4%)	< 0.001	0 (0%)	0 (0%)	
Chronic respiratory illness	40 (6.8%)	22 (5.5%)	18 (9.5%)	0.07	13 (11%)	8 (6.8%)	0.25
Chronic liver disease	11 (1.9%)	10 (2.5%)	1 (0.5%)	0.12	1 (0.8%)	1 (0.8%)	1.00
Malignancy	19 (3.2%)	11 (2.8%)	8 (4.2%)	0.35	7 (5.9%)	5 (4.2%)	0.55
PaO_2_/FiO_2_ ratio (mm Hg)	124 (92–141)	130 (97.5–150)	105.35 (80–134)	< 0.001	123 (81–140)	110.7 (87.1–136)	0.38
Vasopressor	415 (70.3%)	303 (75.8%)	96 (50.5%)	< 0.001	71 (60.2%)	67 (56.8%)	0.60
Sofa score	2 (2–5)	3 (2–5)	2 (1–4)	< 0.001	2 (2–4)	2 (1–4)	0.06
*Laboratory data*							
CRP (mg/L)	163.3 (91.9–246.7)	166.5 (99.6–245.3)	156.2 (69.5–255)	0.29	175.4 (90.9–257)	152 (72–232.1)	0.16
Ferritin (mcg/L)	1057 (637–1625)	1017.5 (638.5–1559)	1118 (638–1942)	0.14	1168.5 (693–1565)	1118.5 (688–1924)	0.27
D-Dimer (mcg/mL)	1.25 (0.68–3.95)	1.35 (0.68–4.23)	1.17 (0.74–3.24)	0.60	1.43 (0.61–4.6)	1.13 (0.74–2.88)	0.99
Platelets (10^9^/L)	233.5 (184–306)	240 (188–312)	221 (176–293)	0.06	249.5 (195–317)	236 (186–308)	0.51
Creatinine (micromol/L)	85 (70–111)	84 (69–110)	93 (71–113)	0.08	78 (64–98)	86 (68–108)	0.07
Bilirubin (micromol/L)	11 (8–15)	11 (8–15)	11 (7–16)	0.74	10 (8–14)	10.25 (7–14.5)	0.95
ALT (IU/L)	40 (25–64)	38 (24–59)	45 (26–70)	0.03	37 (23–64)	45 (28–70)	0.053
AST (IU/L)	51 (35–80)	50 (35–77)	54 (35–91)	0.07	50.5 (36–86)	54.5 (36–91)	0.42
*Corticosteroids*							
Dexamethasone	168 (28.5%)	96 (24.0%)	72 (37.9%)	< 0.001	32 (27.1%)	41 (34.7%)	0.2
Methylprednisolone	446 (75.6%)	331 (82.8%)	115 (60.5%)	< 0.001	88 (74.6%)	79 (66.9%)	0.2
Hydrocortisone	142 (24.1%)	110 (27.5%)	32 (16.8%)	0.005	15 (12.7%)	14 (11.9%)	0.84
Tocilizumab	329 (55.8%)	251 (62.7%)	78 (41.1%)	< 0.001	56 (47.5%)	54 (45.8%)	0.79
Interferon	65 (11.0%)	49 (12.3%)	15(7.9%)	0.12	11 (9.3%)	10 (8.5%)	0.82
*Anti-viral drugs*							
Favipiravir	78 (13.2%)	47 (11.8%)	31 (16.3%)	0.13	17 (14.4%)	23 (19.5%)	0.30
Lopinavir-Ritonavir	306 (51.9%)	229 (57.3%)	77 (40.5%)	< 0.001	58 (49.2%)	51 (43.2%)	0.36
Oseltamivir	355 (60.2%)	267 (66.8%)	88 (46.3%)	< 0.001	63 (53.4%)	58 (49.2%)	0.51
Remdesivir	2 (0.3%)	0 (0%)	2 (1.1%)	0.10	0 (0%)	0 (0%)	
Ribavirin	61 (10.3%)	50 (12.5%)	11 (5.8%)	0.01	9 (7.6%)	7 (5.9%)	0.60

Data are presented as count (%) or median (interquartile range) unless otherwise indicated

Laboratory data and SOFA score were obtained at baseline on ICU admission

BMI, body mass index; PaO_2_/FiO_2_ ratio, ratio of partial pressure arterial oxygen and fraction of inspired oxygen; SOFA score, sequential organ failure assessment score; CRP, C-reactive protein; ALT, alanine transaminase; AST, aspartate aminotransferase

Propensity score matching generated 118 matched sets. Post matching, the two groups did not have any statistically significant differences (Table 1). In the matched cohort, the median time from ICU admission to initiation of IVIG therapy was 6 days (IQR 2.2–11.1 days). The median cumulative dose of IVIG received was 152.0 g (IQR 108.0–235.0 g), and the median number of doses received was 5.0 (IQR 3.0–5.0).

### Outcomes

The all-cause ICU mortality for COVID-19 pneumonia patients admitted with respiratory failure requiring invasive mechanical ventilation for moderate-severe ARDS was 27.1%, and for the matched cohort, it was 25.8%. ICU mortality was significantly higher in the IVIG group (36.4% vs. 15.3% for IVIG and routine care, respectively; sHR 3.5; 95% CI 1.98–6.19; *P* < 0.001). In the propensity score-matched and propensity score-adjusted survival analysis, there was an increased risk of death in the IVIG group compared to the routine care group (Table 2). Results were similar, with an increased risk of death in the IVIG-treated patients for the subgroups based on PaO_2_/FiO_2_ ratio and serum ferritin level. Association of IVIG with increased ICU mortality was significant regardless of the time from ICU admission to IVIG therapy and the number of doses received (Table 3).

**Table 2:** Association of IVIG treatment with mortality

	Total (n)	sHR (95% confidence interval)	*P* value
PS matched analysis	236	3.50 (1.98–6.19)	< 0.001
PS adjusted analysis	556*	2.98 (1.92–4.60)	< 0.001

IVIG, intravenous immunoglobulin; PS, propensity score; sHR, sub-distribution hazard ratio

*Propensity score could not be calculated for 34 patients due to complete separation by baseline characteristics

**Table 3:** Association of IVIG treatment with mortality among sub-groups

Sub-groups	sHR (95% confidence interval)	*P* value
*PaO*_*2*_*/FiO*_*2*_* ratio*		
> 100 mm Hg	3.44 (2.04–5.78)	< 0.001
≤ 100 mm Hg	2.17 (1.07–4.41)	0.03
*Serum ferritin level*		
< 1000 mcg/L	3.45 (1.87–6.38)	< 0.001
≥ 1000 mcg/L	2.79 (1.52–5.11)	0.001
*Time from ICU admission to IVIG therapy*		
< 5 days	2.65 (1.07–6.56)	0.035
≥ 5 days	3.89 (1.85–8.18)	< 0.001
*Number of IVIG doses received*		
≤ 3 doses	3.72 (1.39–9.91)	0.009
> 3 doses	3.42 (1.69–6.93)	0.001

IVIG, intravenous immunoglobulin; sHR, sub-distribution hazard ratio; PaO_2_/FiO_2_ ratio, ratio of partial pressure arterial oxygen and fraction of inspired oxygen; mcg/L, microgram per liter

Compared to routine care group, ventilator-free days at day-28 were lower in the IVIG group [median (IQR); 0 (0–18) vs. 22 (7–24) days; *P* < 0.001], and ICU-free days at day-28 were also lower in the IVIG group [median (IQR); 0 (0–8.8) vs. 16 (0–20) days; *P* < 0.001]. Additionally, the incidence of AKI was significantly higher in the IVIG group (85.6% vs. 67.8% for IVIG and routine care, respectively; *P* = 0.001).

## Discussion

Our single-center retrospective study revealed a significant association of IVIG therapy with higher ICU mortality in patients with COVID-19 pneumonia receiving invasive mechanical ventilation for moderate-to-severe ARDS. We confirmed these results after propensity score matching to account for the differences between the two groups.

Only a few randomized controlled studies have been conducted to evaluate the efficacy of IVIG therapy in COVID-19 pneumonia. Gharebaghi et al. have reported that administration of IVIG to 30 patients with severe COVID-19 infection who did not respond to initial treatment significantly reduced the in-hospital mortality (20.0% in the treatment group vs. 48.3% in the control group; *P* = 0.025). However, the authors did not report the severity of ARDS and the percentage of patients receiving invasive mechanical ventilation [14]. Another pilot randomized controlled trial showed that IVIG 0.5 g/kg daily for three days with concomitant methylprednisolone 40 mg significantly improved hypoxia and reduced progression to mechanical ventilation in COVID19 patients [15]. The clinical improvement found in this study cannot be generalized because of the small sample size and concomitant use of methylprednisolone therapy that may have confounded the results. Recently, Tabarsi et al. demonstrated that IVIG in combination with hydroxychloroquine and lopinavir/ritonavir for SARS-CoV-2 patients did not reduce mortality or need for mechanical ventilation, and did not improve radiological findings. However, the potential benefit of IVIG monotherapy couldn't be evaluated in this study due to the use of combination therapy with hydroxychloroquine and lopinavir/ritonavir [16].

The novelty of our work comes from exclusively studying critically ill COVID-19 pneumonia patients receiving invasive mechanical ventilation for moderate-to-severe ARDS. Based on a global literature survey, the mortality rate in COVID-19 associated ARDS was 45%, and the incidence of ARDS among non-survivors of COVID-19 was 90%. In the same study, the mortality rate of patients who received invasive mechanical ventilation was 59% [17]. Overall ICU mortality of our patients with moderate-to-severe ARDS receiving invasive mechanical ventilation was lower (27.1%). This could be attributed to the younger age of our population (median age 53 years, Table 1) and is consistent with previous reports of higher mortality with increasing age [18].

The higher mortality observed in our patients who received IVIG could be related to multiple factors. Firstly, we observed a higher incidence of AKI in the IVIG group, and based on a recent report, the occurrence of AKI had increased the risk of death by 60% in patients with COVID-19 [19]. Secondly, IVIG was administered after ICU admission and receiving invasive mechanical ventilation for moderate-to-severe ARDS, which is late in the course of the disease. The median time from ICU admission to IVIG therapy was 6.28 days in our study. Xie et al. has reported that IVIG therapy for COVID-19 pneumonia within 48 h of ICU admission improved clinical outcomes [20]. Thirdly, thromboembolic events are high in SARS-CoV-2 infected individuals with significantly increased odds of mortality [21]. Also, previous studies have suggested an association between IVIG and increased risk of thromboembolic events [22, 23]. Hence, initiating IVIG therapy in patients with predictive factors for thrombotic complications could have resulted in worse clinical outcomes in our cohort. Lastly, though the control and treatment groups were matched based on their baseline characteristics (including PaO_2_/FiO_2_ ratio and SOFA score) at ICU admission, IVIG was administered mainly by physicians as salvage therapy to deteriorating patients who had not responded to initial management.

The dose and duration of IVIG administered to our patients were based on established practice in immune modulation therapy for other diseases [24], as there are no standardized guidelines for its use in COVID-19 patients. In a retrospective case series of 12 patients where IVIG appeared to improve the clinical course, the total dose administered ranged from 0.5 to 2.0 g/kg (median 1.25 g/kg) distributed over 1–4 daily doses [25]. Further studies are required to investigate the dose, duration, and appropriate time for initiation of IVIG therapy which might benefit COVID-19 patients without having adverse effects.

Our study has few limitations. We used propensity score matching to adjust for known confounders, but due to the retrospective design, we could not entirely exclude the possibility of unmeasured confounding factors that might have led to worse outcomes in the IVIG group. The study was conducted in a single center in Qatar, thereby limiting the generalization of these results to other institutions. Our hospital was a tertiary-care referral center for COVID-19 in the State of Qatar, receiving patients from numerous other healthcare facilities. Despite being rigorous in our data collection and analysis approach, there were missing data for some variables that could have affected the outcomes. We did not define the phenotype of our patients as hypo- or hyper-inflammatory based on the severity of systemic inflammation [26] and hence could not study the role of IVIG in modulating the immune response in hyper-inflammatory COVID-19 ARDS.

## Conclusions

The results of our single-center retrospective study revealed a higher ICU mortality rate, lesser ventilator-free days and ICU-free days at day-28, and higher incidence of AKI in mechanically ventilated patients who received IVIG therapy for COVID-19 related moderate-to-severe ARDS. Despite the limitations, this study highlights the possibility of unfavorable outcomes with IVIG therapy in SARS-CoV-2 infected patients. A multicenter, randomized clinical trial is warranted to investigate further IVIG's efficacy and safety in critically ill COVID-19 patients.

## Supplementary Information

**Additional file 1.** Anticoagulation protocol for critically ill COVID-19 patients at Hazm Mebaireek General Hospital, Qatar.

## Data Availability

The datasets generated and/or analyzed during the current study are available from the corresponding author on reasonable request.

## Abbreviations

AKI: Acute kidney injury
ARDS: Acute respiratory distress syndrome
COVID-19: Coronavirus disease 2019
CRP: C-reactive protein
ICU: Intensive care unit
IVIG: Intravenous immunoglobulin
PaO_2_/FiO_2_: Ratio of partial pressure arterial oxygen and the fraction of inspired oxygen
SARS-CoV-2: Severe acute respiratory syndrome coronavirus 2
SOFA: Sequential organ failure assessment
