# Supplementary material for: Clinical outcomes of intravenous immunoglobulin therapy in COVID-19 related acute respiratory distress syndrome: a retrospective cohort study
Source: BMC Pulm Med. 2021 Nov 8;21:354. doi: 10.1186/s12890-021-01717-x (PMC8572690; doi:10.1186/s12890-021-01717-x)
Supplement: Supplementary file 1 — Additional file 1. Anticoagulation protocol for critically ill COVID-19 patients at Hazm Mebaireek General Hospital, Qatar. [file 12890_2021_1717_MOESM1_ESM.docx]

**Figure A1. Anticoagulation protocol for critically-ill COVID-19 patients at Hazm Mebaireek General Hospital, Qatar.**

**
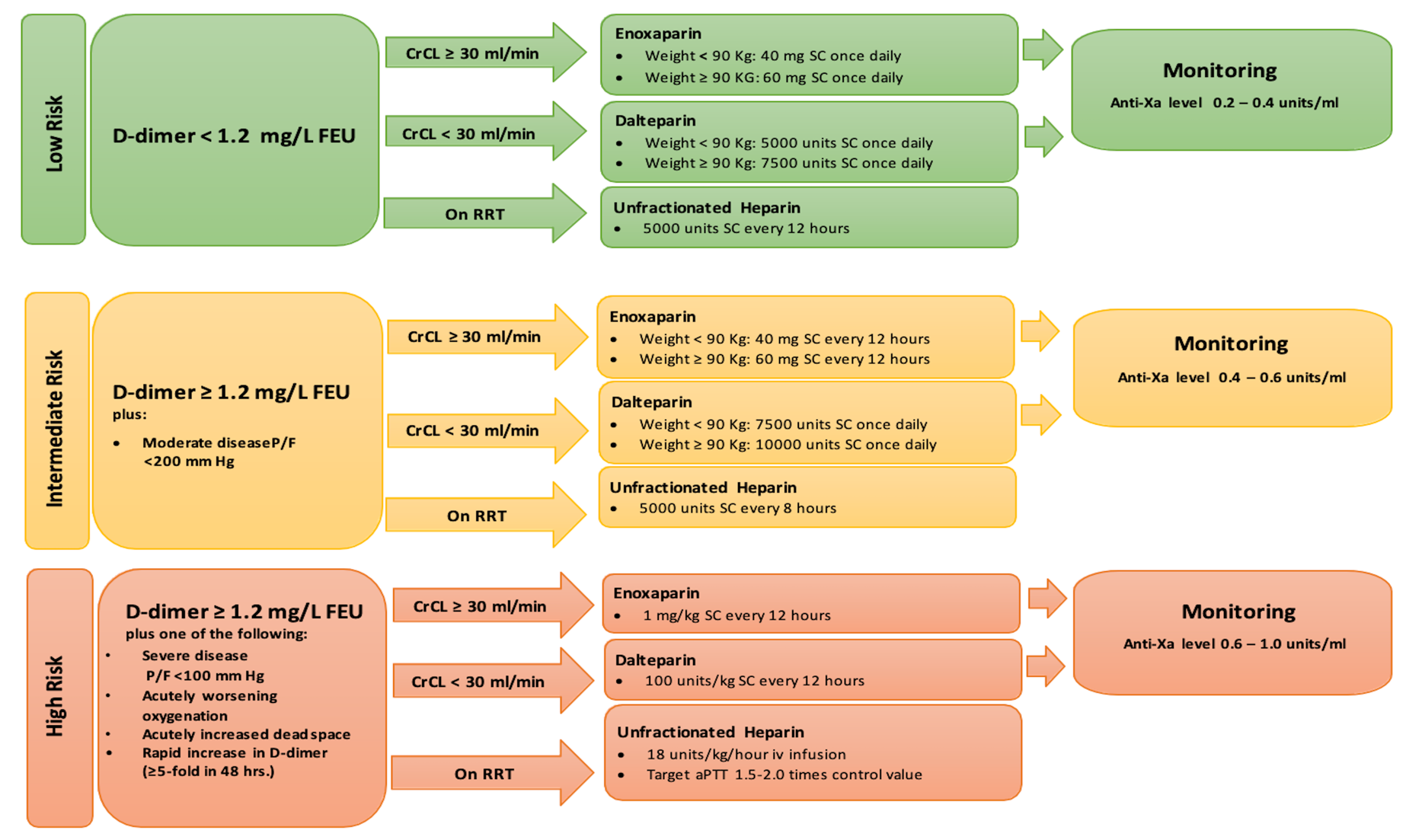
**

D-dimer < 0.46 mg/L (milligram/liter) FEU (fibrinogen-equivalent units) is considered negative.

P/F: ratio of partial pressure arterial oxygen and fraction of inspired oxygen, CrCL: creatinine clearance, RRT: renal replacement therapy, SC: subcutaneous, aPTT: activated partial thromboplastin time, Anti-Xa: anti-factor Xa assay.
